# Adrien Proust – overlooked as an occupational health practitioner, author and teacher

**DOI:** 10.1186/s12995-026-00508-6

**Published:** 2026-03-21

**Authors:** Kjell Torén, Carl Lindgren, Paul D. Blanc

**Affiliations:** 1 Occupational and Environmental Medicine, School of Public Health and Community Medicine, Sahlgrenska Academy, University of Gothenburg, Gothenburg, Sweden; 2 Department of Occupational and Environmental Medicine, Sahlgrenska University Hospital, Västra Götalandsregionen, Gothenburg, Sweden; 3 Swedish Society of Marcel Proust, Stockholm, Sweden; 4 Swedish Medical Society for the History of Medicine, Stockholm, Sweden; 5 Division of Occupational, Environmental and Climate Medicine, Department of Medicine, University of California, San Francisco, CA US; 6 School of Public Health and Community Medicine, Sahlgrenska Academy, University of Gothenburg, Box 414, Gothenburg, SE-405 30 Sweden

**Keywords:** History of Medicine, WHO, Cholera, Byssinosis, Women, Night work

## Introduction

Publications touching Dr. Adrien Proust (1834–1903) often focus on his son, Marcel Proust (1871–1922), a famous author [1]. Such papers examine the biography of Adrien Proust for better understanding of the environment in Marcel Proust’s famous novel In Search of Lost Time, sometimes also translated as Remembrance of Things Past [*À la recherche du temps perdu*]. Other aspects of Adrien Proust’s work have been recognized by others but nonetheless warrant revisiting [2–4]. First and foremost, Adrien Proust has been overlooked as a public health activist, and in particular his role in occupational medicine largely has been forgotten. He was also the author of multiple books intended to educate the French population in public health and hygiene. These activities of Adrien Proust were combined with teaching and administrative acumen. In his own time, Proust was widely acknowledged for his negotiating skill during international sanitary conferences, especially for his handling of contentious British delegates.

## Methods

For this paper we used multiple resources for information. The two main sources were PubMed to identify relevant scholarly articles, and the sole biography of Adrien Proust written by Daniel Panzac [*Le Docteur Adrien Proust. Père méconnu*,* précurseur oublié*] [3]. The text of the biography is in French, and all French translations for this paper were made by a single author (KT). That biography is also the source for a comprehensive bibliographic list of Proust’s publications. We also closely reviewed Proust’s key occupational texts, especially *Traité d’hygiène publique et privée* in all three of its editions.

### Early life and training

Adrien Proust was born 1834 in Illiers outside Chartres, France. **His** parents were shopkeepers. He was a brilliant student and went on to receive his medical degree (MD) in 1862 at the Medical Faculty [*Academie de medicine*] at University of Paris submitting a dissertation on idiopathic pneumothorax [*Du pneumothorax essential ou pneumothorax sans perforation*] [5]. Up until that time, the received medical wisdom had been that in absence of a rupture permitting air from the lungs to enter the pleura, pneumothorax developed through transformation of pleural fluid into gaseous matter. By presenting 25 cases, Proust convincingly argued that the cause is always incoming air even if it may be difficult to detect the site of the leak. Through this innovative analysis early in his career, he demonstrated astute critical thinking.

Four years later in 1866, Proust presented his PhD thesis on different forms of stroke and their associations with various diseases [*Différentes formes de ramollissement du cerveau*] [6]. Based on this thesis, he received qualification to teach at the university [*professeur agrégé*] [6]. This was the same year (1866) when Paris was struck by a cholera outbreak. At that time, Proust was chief physician in the Departments of Medicine and Pathology at the Charité Hospital in Paris.

### Cholera in Paris and Europe

Cholera came to Europe for the first time in 1829 when an outbreak occurred in Orenburg in southeast Russia after a slow spread of transmission from India over the steppes. It then spread rapidly to Novgorod, then Moscow, and shortly later to the rest of Europe.

At this time the medical profession had no idea as to how cholera spread. Moreover, there was no cure. In his clinical duties, Adrien Proust experienced how his patients died rapidly without any cure. He became convinced that the disease could only be treated effectively through with prevention. He likely was influenced in this thinking by Antoine Fauvel (1813–1884), a senior colleague who was a strong supporter of quarantines and who later was the State epidemiologist (Surgeon general) [*Inspecteur général des Services sanitaires*] in France. Fauvel proposed to the imperial French government that Adrien Proust should be sent out on an expedition to explore the mode of cholera transmission. That 1869 expedition has been well described; Proust’s main conclusion was that cholera had been spread from India via Egypt, where the pilgrimages to and from Mecca played a very important role [3, 7].

This experience was scientifically formative for Proust. Hygiene, which included occupational medicine and various environmental topics, was an expanding field of medical endeavor in the nineteenth century. At that time, France was a leader in this field. Indeed, the first scientific journal in this field was the *Annales d’hygiène publique et de médecine légale*, published from 1829 in Paris [8]. From 1869 and onward, Adrien Proust devoted himself to the prevention of disease.

## Author and occupational health practitioner

Even by today’s standards, Adrien Proust authored an impressive body of work, even more so considering that he composed with pen and paper and without electrical lighting. During the period 1860 to 1903 he produced 201 publications as well as multiple full-length monographs [3] (Table 1). Reviewing these publications over time it becomes clear that his focus shifted from clinical medicine to occupational and public health. From the years 1860 to 1869. he authored one (7%) publication in occupational and public health. The proportion increased to 46% (1870–1879), 68% (1880–1889), 94% (1890–1899) and to 100% his final years of output (1900–1903). The period 1880–1889 was the most productive, with 99 publications [3]. This rapid evolution in his interests from narrow clinical medical topics to broader questions of population health, occupational medicine and hygiene are also reflected in his published books. These are listed in the Table. In addition to these publications, he was also an active research supervisor. Panzac reports that during 1886–1903, there were 70 theses from the Medical Faculty in Paris covering the topic hygiene of which 24 were supervised by Proust [3].

**Table 1:** Books and Monographs by Adrien Proust derived from Panzac [3]

Title	Year of publication	No of pages
Du pneumothorax essentiel sans perforation	1862	54
Des differents formes de ramollisement du cerveau	1866	134
Essai sur l’hygiène internationale	1873	421
Traité d’hygiène publique et privée, 1st edition	1877	840
Traité d’hygiène publique et privée, 2nd edition	1881	984
La choléra: étiologie et prophylaxie	1883	232
Douze conférences d’hygiène	1890	182
La défense de l’Europe contre le choléra	1892	454
L’hygiène des expeditions coloniales	1895	23
L’hygiène du goutteaux	1896	338
L’orientation nouvelle de la politique sanitaire	1896	456
L’hygiène de l’obèse	1897	344
L’hygiène du neurasthénique	1897	282
La défense de l’europé contre la peste et la conférence de Venise de 1897	1897	452
L’hygiène de du diabétique	1899	283
Organisation de la défense sanitaire du port de Marseille	1899	11
Traité d’hygiène publique et privée, 3rd edition	1902	1247

The book that made Adrien Proust famous as a leading hygienist, including occupational health, was his 1877 *Traité d’hygiène publique et privée* [9]. This, the first edition of the text, contains 840 pages divided into 14 chapters. At that time, several other comprehensive textbooks in hygiene were extant. The unique feature of Proust’s textbook was its scientific method: he based the text on, for that time, modern biomedical knowledge, citing many references from contemporary authorities to support his conclusions. He was less focused on theories and more prone to cite observational studies. The textbook was well appreciated by his contemporaries, and he produced an enlarged second edition in 984 pages, only four years later in 1881. Near the end of his life, in 1902, a third very extended edition (1247 pages) was published (Table).

In the first edition of *Traité d’hygiène publique et privée* a subsection on occupational risk factors (*des professions*), comprises 370 pages and accounts for the bulk of the book’s 416-page third section, *De l’homme consideré comme individu.* In this section, Proust describes in detail different occupational diseases caused by chemicals and metal dust, as well as discussing physical workload and characterizing the *rétraction de l’aponévrose palmaire*, (Dupuytrens disease) among workers whose hands are exposed to pressure and repeated sudden impacts (*chocs brusque*). Of note, this was at a time when pneumatic tools had not yet been invented. Proust especially mentions coachmen, fencing instructors and polishers.

The text dealing with respiratory tract diseases highlights different occupations carrying increased risk for chronic bronchitis, emphysema and pneumonia. Proust gives special attention to a condition that, thanks to Proust, came to be known as byssinosis. He points out that an authority of occupational diseases at that time, Ludwig Hirt (1830–1913) had misinterpreted the term Greek word” lyssinos”, thus coining in error a term for cotton dust-induced lung disease [10]. Proust noted on page 171 of the first edition that Hirt wrongly writes Lyssinosis. By a singular inadvertence the author confuses λύσσα, rage (rabies), with βὑσσος, cotton “[Cést à tort que Hirt écrit Lyssinosis. Par une singulière inadvertance l’auteur confond λὑσσα, rage avec βὑσσος, coton] “ [9, 11].

Proust authored an enlarged second edition (984 pages) in 1881, only four years after his first and with little change in its occupational content. The third and final edition, published the year before Proust’s death, was considerably extended. Proust engaged two younger colleagues, that also had been his students, as co-authors: Arnold Netter (1855–1936), a pediatrician and bacteriologist, and Henri Bourges (1861–1932), head of the Hygienic laboratory in Paris. Proust knew his co-authors from their common work as members of the *Comité consultative d’hygiene*, which was group giving advice to the Secretary of Interior. These two co-authors reflect the needs of additional competence regarding the newly obtained knowledge emerging about microorganisms. In the third edition, the section devoted to occupational medicine and its risk factors were less prominent relatively and in absolute terms, comprising only 181 pages out of 1247 pages, and the chapter was moved to be the last of the text.

There were, however, several new subsections in the occupational chapter. It is unlikely that Proust’s co-authors had a hand in these updates. For example, new material concerned the prevention of dust induced diseases, describing various methods of achieving this, accompanied by illustrations. The principal method Proust promoted was use of facemasks, but he also strongly stressed the importance of the workplace changes, that would be recognized today as taking precedence in the hierarchy of controls, such as enclosing machinery and using and using water to dampen down dust. It is clear from new material that Proust kept current with emerging medical knowledge on occupational disease. One example is that he wrote about Thomas slag pneumonia, a pneumonia that occurred among workers exposed to iron and manganese when they extract phosphorous from the alkaline slag [12]. This was described from France in 1888, and Proust cites this work [13]. He also mentions bladder cancer among aniline workers, which was first reported by Rehn in 1895 [14]. The third edition carries forward from the previous edition concerns over adverse effects from the inhalation of various fiber dusts, such as among hemp workers, an area of ongoing interest for Proust based on thesis he supervised [15]. Regarding cotton fiber byssinosis, the text about Hirt and the misnaming of lyssinosis was deleted.

Proust also included new text about occupationally induced vocal problems among actors and singers. This is notable because Proust also served as a consulting physician for the staff at *l’Opéra-comique*. As such, he had experienced different occupational problems in the staff; he was also supervisor for a doctoral thesis about muscular and tendonous strains among dancers [16].

Another topic area with extensive updated text in the third edition addresses occupational and environmental lead intoxication. Proust describes, at page 1161, a baker in Paris who bought old discarded painted wood for use in his owen resulting in heavy contamination of lead in his bread. This resulted in several cases of lead poisoning among his customers in the quarters close to the bakery. Proust also lists thirteen occupations with high risks for lead poisoning, including paint manufacturing workers, tin founders, polishers, munitions workers, building painters and typographers. He ends this subsection with preventive advice; reducing contact with lead; in paints, replacing lead with zinc; improving ventilation in the workplace; and washing of the hands, face and mouth. In addition to this comprehensive subsection about lead, Proust also authored several scientific publications about lead intoxication as well as supervising doctoral theses about lead intoxication [17–19].

As a practitioner and governmentally appointed *Inspecteur général des Services sanitaires* (from 1884), Proust probably encountered patients with occupational diseases. This helps explain why occupational diseases hold such a prominent place in *Traité d’hygiène publique et privée*. In addition, in several publications in 1881 (as well as in *Traité d’hygiène publique et privée*), Proust paid attention to the health hazards in coal mining [20–21]. He described breathing problems, accidents, fractures and burns, but he also noted improvements in the mines; the ladders were replaced by mine elevators, mechanical fans were introduced and there were also improved conditions for the horses pulling the mine wagons. The poor working conditions in the coal mines, also the awful conditions for the mine horses, were also noted by Emile Zola, in his famous 1884 novel, *Germinal*. There is no evidence that Emile Zola was directly influenced by Proust’s work, but it does underscore that the coal workers’ occupational environment was viewed critically by leading authorities from different disciplines.

In one his major engagements in the field of occupational medicine, Proust asserted in a lecture in *l’Académie des Sciences morales et politique* that France must restrict night work for women [22]. In his hospital practice in Paris, Proust had encountered women, especially pregnant women, working night shifts. He noted a high occurrence of anemia and tuberculosis among these women and premature death among their offspring, and Proust argued that these observations were also confirmed in an investigation of silk workers in Lyon. This differed from his previous papers about occupational diseases, as this time he also proposed an intervention. Proust cited a Swiss law from 1877 that banned work from women on Sundays and at nights and went so far as to mandate eight-weeks maternity leave, at that time a nearly revolutionary intervention. Proust proposed in his lecture in *l’Académie des sciences morales et politiques* that France should implement the same rules [22–23]. He was heavily criticized by established economists for his views on night work among women. In particular, Léon Say (1826–1896), a noted economist and the French Minister of Finance from 1872 to 1883, pointedly argued that hygienists like Proust should deal with the air, water and microbes but they should not express opinions about the laws that rule society [22]. This conflict resurfaced ten years later, see below. Adrien Proust likely was very engaged with his patients with occupational disease. This is consistent with the way his grandchild, Suzy Mante-Proust, categorized three aspects of her grandfather: his neurological research; his teaching; and his work with occupational diseases [24].

Of note, his son, Marcel, may have been inspired to illustrate his father’s work in his novel. In the first volume, Du côté de chez Swann, of the *À la recherche du temps perdu* there is a description of a case of occupational asthma. On page 105 (French edition) it portrays a scullery maid who has recurrent attacks of asthma brought on by peeling asparagus [25]. The chief kitchen maid vindictively assigns her additional work with the vegetables. Occupational asthma due to asparagus is a well described entity with immediate asthmatic symptoms after bronchial challenge with raw asparagus [26].

### General public hygiene

Beyond biomedical writing, Adrien Proust also assumed the role of a public health activist, reflected in many of his later publications that took the form of popular science writing with the explicit aim of educating the public in hygiene and lifestyle, a teaching aspect of his work. The books he authored and edited in the series *L´Hygiene* (1895–1899) provide a prime example (Table). Even earlier during 1881–1882 when France launched mandatory public schooling, Proust promoted the view that all pupils should be educated about contagious diseases, personal hygiene and healthy eating habits [3]. From 1881 to 1885, he was also a college teacher in hygiene at *École normale supérieure*, in Fontenay outside Paris.

In 1885, at age 56, Proust was appointed as Professor in Hygiene at *Académie de Médecine* in Paris. As a university professor he ran courses in hygiene, that also included students from the well-known *l’Institut Pasteur*. He introduced visits at workplaces in his teaching, and the students visited local factories, slaughterhouses, hospitals, and building sites including sewage construction [3].

### Cholera and the international sanitary conferences 1851–1903

Proust was to play a major role in international quarantine policies. Since earlier in the nineteenth century, France had tried to organize meetings that might lead to international quarantine agreements. The British presented a major stumbling block to successfully reaching such accords, taking a negative position on any rules that they perceived might hinder their international trade. In 1851, when representatives of eleven countries finally did meet in Paris on this subject, Great Britain, (joined by Austria) took the position that quarantines were useless for cholera protection, citing a rationale that the disease was spread through the general atmosphere and arguing further that it only affected poor and already diseased persons [27]. The following meetings, 1859–1874, were mainly without substantive results. The 1874 meeting in Vienna was the first in which Adrien Proust participated.

The sixth conference was held in 1885 in Rome, in the aftermath of the detection of the vibrio bacillus. Proust again was one of the French delegates, and Koch was one of the German delegates championing the position that cholera was spread via drinking water. Some conference delegates remained skeptical, especially the British, who rejected all notions that cholera might be spread via ships from India to Europe, blocking any “theoretical discussions on the aetiology of cholera” [28].

Right through to the next meeting in 1892 (held in Venice), the British delegation continued in its resistance to quarantines [29]. The French delegation, with Proust, Camillle Barrère (1851–1940), and Paul Brouardel (1837–1906), demanded a five-day quarantine in Egypt for ships with any suspected cholera onboard. After days of negotiations, Adrien Proust drafted a proposal that the ships should be divided into three classes: *navires indemnes* (clean), *navires suspects* (cholera onboard but no new cases noted over the last seven days) and *navires infectés* (new cases within the last seven days). The first group was free to sail away, while the other two groups would be forced to different lengths of quarantine. The document was initially blocked by Britain, but after a subsequent meeting in Paris the same year, all participating countries finally did come to ratify this proposal [27]. Indeed, this was the first international agreement of any kind in the field of public health. As the leader in bringing this to successful fruition, this would have been a personal triumph for Adrien Proust, but more importantly, it was a key multinational victory over disease.

The 11th International Sanitary Conference was held in Paris from 10 October to 3 December 1903, and this was the last sanitary conference that Adrien Proust attended. By then he was an international celebrity in the field, delivering the keynote address in which he summarized what had transpired in more than half a century of such meetings [30]. He ended by emphasizing the need of establishing a standing international sanitary bureau in Paris, with French as its working language [27]. The meeting also decided, also as proposed by Proust, to merge all previous agreements into a single international public health treaty, and that the French government should establish an international health office at Paris [27]. Sadly, Proust did not live to see the final ratification of this plan, succumbing to a stroke the week before these decisions were taken, 26 November 1903.

In November 1908, the *Office International d´Hygiène Publique* was inaugurated in Paris and remained there for the coming four decades until superseded by World Health Organization (WHO).

That Proust was an international authority in cholera even was touched upon by Gabriel Garcia Marquez in his acclaimed novel Love in the Time of Cholera [*El amor en los tiempos del cólera*]. The novel’s protagonist, a medical doctor named Juvenal Urbino, seeks to learn all there is to know about different forms of cholera. In so doing, he becomes a disciple of the most prominent epidemiologist of the time and the creator of the isolation zones against the spread of cholera, Professor Adrien Proust.

### Final years

For quite some time, Adrien Proust had wished to be elected as a member of *Institute de France*. This institute organized the five French scientific academies, among others the *Académie Française*,* Académie des sciences*, and *Académie des sciences morales et politiques.* Proust applied to the latter and not the former, consistent with his more prominent role as a public health activist and policymaker, rather than a medical researcher. He was twice unsuccessful in seeking election, in 1898 and 1901. His election was resisted by the conservative group of economists, who still were annoyed over that he in 1890 argued for a law that restricted night work for women [3]. Instead, an economist and an historian, respectively, were elected in those years. He did not live long enough to overcome this opposition.

**Fig. 1:**
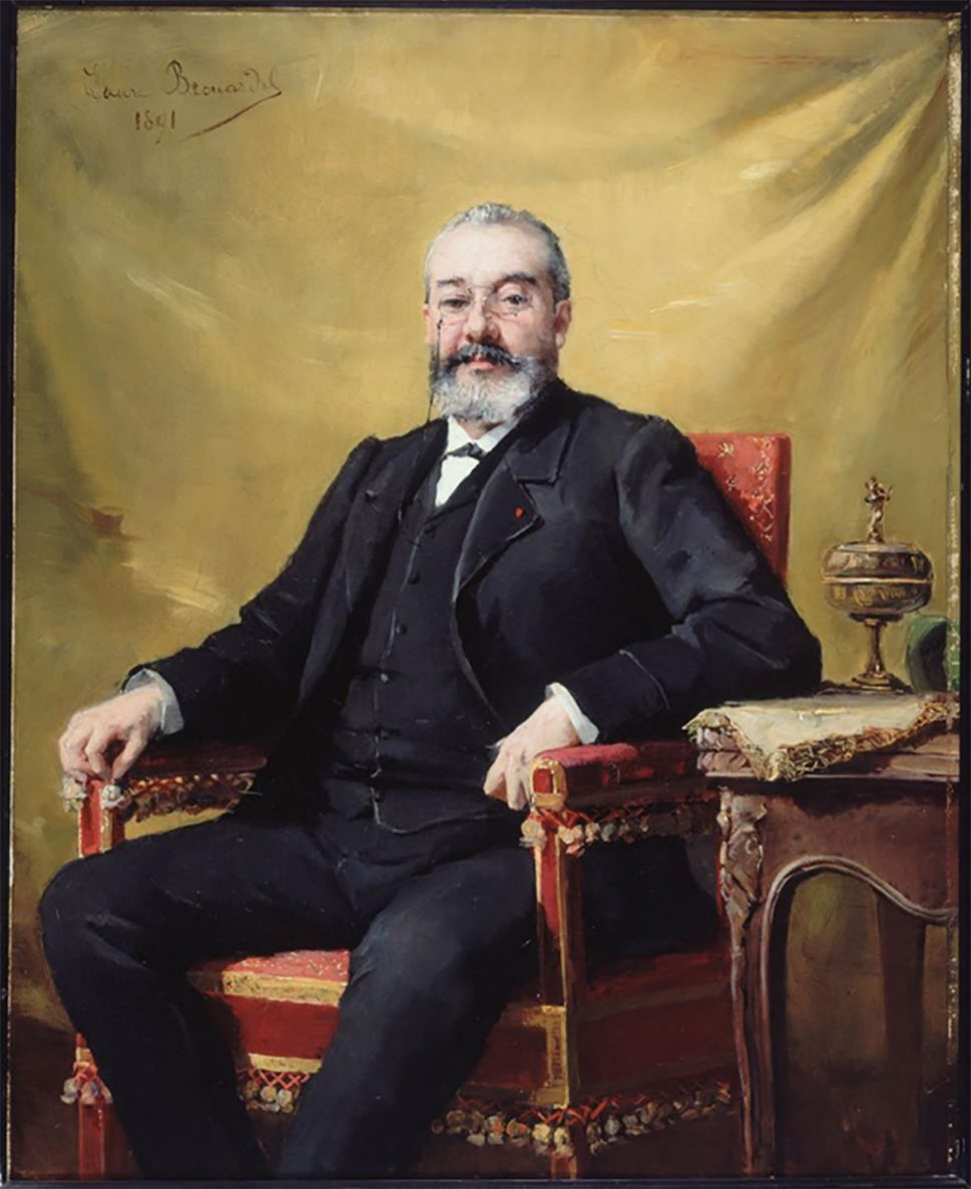
Portrait of Adrien Proust painted 1891 by Laure Brouardel (1852–1935), wife to Adrien Proust’s lifelong colleague Paul Brouardel. Donated 1973 to Musée Carnavalet, Paris, by Jacques Guérin. Downloaded Jan 24th, 2026, from https://images.google.com

During decades, Proust worked very closely with Camillle Barrère, a diplomate, and with Paul Brouardel, dean of the medical faculty in Paris. Laure Brouardel (1852–1935), the wife of Paul Brouardel, made 1891 a well-known oil painting of Adrien Proust (Fig. 1).

## Conclusions

Adrien Proust was an outstanding occupational and public health physician. His role as a leader in occupational medicine has been insufficiently recognized. He was an important author of biomedical works aimed at educating medical professionals as well as a wider French readership in public health and hygiene. He enjoyed great success putting his knowledge into practice. He was instrumental in the progress of the International Sanitary Conferences from 1864 to 1903, work that implemented preventive measures against cholera. This work served as one of the templates for the later international actions arguably as one of the forerunners of today’s WHO. He has been recognized in that regard, but his important role in occupational health is less well appreciated.

## Data Availability

No datasets were generated or analysed during the current study.
